# The Landscape of PDK1 in Breast Cancer

**DOI:** 10.3390/cancers14030811

**Published:** 2022-02-05

**Authors:** Na Wang, Jianjiang Fu, Zhihua Li, Ningni Jiang, Yanhong Chen, Juan Peng

**Affiliations:** 1 Department of Pathology, Key Laboratory of Reproduction and Genetics of Guangdong Higher Education Institutes, Key Laboratory for Major Obstetric Diseases of Guangdong Province, The Third Affiliated Hospital of Guangzhou Medical University, Guangzhou 510150, China; wangna10197@126.com (N.W.); joe2042632928@outlook.com (J.F.); 2011683132@gzhmu.edu.cn (Z.L.); jiangningni@stu.gzhmu.edu.cn (N.J.); 2011683122@gzhmu.edu.cn (Y.C.); 2 The Third Clinical School, Guangzhou Medical University, Guangzhou 510150, China; 3 Department of Fetal Medicine and Prenatal Diagnosis, The Third Affiliated Hospital of Guangzhou Medical University, Guangzhou 510150, China; 4 Department of Obstetrics and Gynecology, The Third Affiliated Hospital of Guangzhou Medical University, Guangzhou 510150, China

**Keywords:** PDK1, breast cancer, survival, prognosis, targeted therapy

## Abstract

**Simple Summary:**

Approximately 2,261,419 new cases of breast cancer (BC) and 684,996 BC-related deaths are estimated to occur in 2020 globally. New individualized therapeutic strategies are urgently needed for affected patients. The aim of our review was to assess the potential role of targeted PDK1 therapies in BC. We hope the information provided on clinical trials of PDK1-targeted therapies will benefit researchers and clinicians in the breast cancer field.

**Abstract:**

Given that 3-phosphoinositide-dependent kinase 1 (PDK1) plays a crucial role in the malignant biological behaviors of a wide range of cancers, we review the influence of PDK1 in breast cancer (BC). First, we describe the power of PDK1 in cellular behaviors and characterize the interaction networks of PDK1. Then, we establish the roles of PDK1 in carcinogenesis, growth and survival, metastasis, and chemoresistance in BC cells. More importantly, we sort the current preclinical or clinical trials of PDK1-targeted therapy in BC and find that, even though no selective PDK1 inhibitor is currently available for BC therapy, the combination trials of PDK1-targeted therapy and other agents have provided some benefit. Thus, there is increasing anticipation that PDK1-targeted therapy will have its space in future therapeutic approaches related to BC, and we hope the novel approaches of targeted therapy will be conducive to ameliorating the dismal prognosis of BC patients.

## 1. Introduction

An estimated 19,292,789 new cancer cases and 9,958,133 cancer-related deaths occurred in 2020 worldwide, increasing the global cancer burden. Apparently, breast cancer (11.7% of the total new cases, BC) took the place of lung cancer (11.4%, LC) for the first time and became the most frequently occurring cancer worldwide. Meanwhile, it ranked the fifth among causes for human cancer-related death globally in 2020 (684,996 deaths, 6.9%) (https://gco.iarc.fr/, accessed on 1 September 2021). In China, BC ranked fourth (416,371 new cases, 9.1%) in the top 10 most frequently occurring cancers and seventh in cancer-related deaths (117,174 deaths, 3.9%), respectively, in this globally recognized list in 2020. In the 2019 World Health Organization (WHO) Classification of Breast Tumors, BC constitutes a series of heterogeneous groups of tumors with marked variations in clinical presentation, biological behavior, and response to therapy [1]. With the development and progression of molecular assays, the classification and management of BC are not only based primarily on clinicopathologic characteristics (morphology, size, grade, nodal status, etc.) and immunophenotypic profile (principally, estrogen receptor (ER), progesterone receptor (PR), and human epidermal receptor 2 (HER2)) but also depend on molecular classifications (*HER2* amplifications or mutations, *ESR1* mutations, *CDH1* somatic mutations, *BRCA1* and *BRCA2* mutations, *GATA3* somatic mutations, *TP53* somatic and germline mutations, *PIK3 CA* and *MAP3 K1* somatic mutations, *ETV6*-*NTRK3* and *MYB*-*NFIB* gene rearrangements, etc.) [1,2].

**Figure 1:**
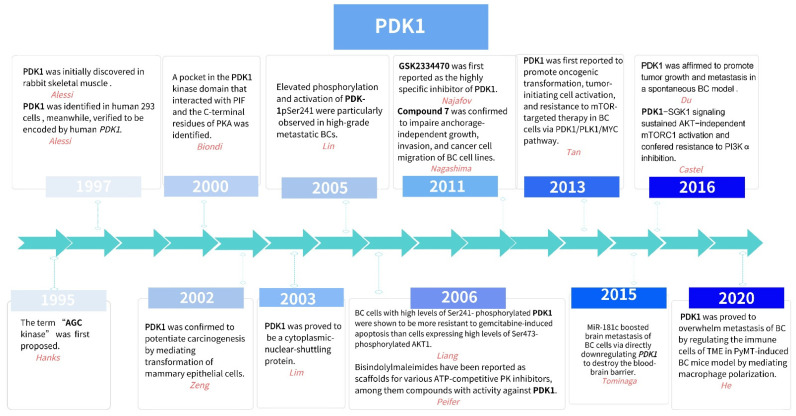
Timeline of the hallmarks in PDK1 pathway research in breast cancer.

Since the term “AGC kinase” was coined by Hanks et al. in 1995 [3] (shown in Figure 1), the AGC kinase family has attracted great attention due to its therapeutic potential in clinics [4]. As a major kinase of the AGC protein kinase family, 3-phosphoinositide-dependent protein kinase 1 (PDK1) has played crucial roles in various cellular process via the PI3 K/AKT-dependent or PI3 K/AKT-independent pathway in BC cells, such as tumorigenesis, growth and survival, metastasis, tumor microenvironment (TME) regulation, and drug resistance. Targeted therapies of PDK1 signaling in BC have been proposed to improve efficacy or even reverse treatment failure.

In this paper, we search the literature from PubMed (https://pubmed.ncbi.nlm.nih.gov/, accessed on 1 September 2021) and review the remarkably short but substantial history of PDK1 in BC to improve the options of individualized therapy for patients with BCs.

## 2. The PDK1 Structure and Pathway

The AGC kinase family contains at least 60 members among 21 subfamilies (AKT, CRIK, DMPK, GRK, LATS, MAST, MRCK, MSK, NDR, PDK1, PKA, PKC, PKG, PKN, PRK, ROCK, RSK, S6K, SGK, SGK494, and YANK), all of which have analogous structures and similar activation manners with respect to two highly conserved motifs: the activation region (referred to as the T- or activation loop), located in the catalytic kinase domain, and the hydrophobic motif (HM), located in a non-catalytic domain [5]. In addition, a third phosphorylation site (the turn motif or zipper site, TM/Z) exists in some AGC kinases, which supports their activation both by stabilizing the HM in its kinase-activating binding site and by protecting the HM from dephosphorylation [6].

**Figure 2:**
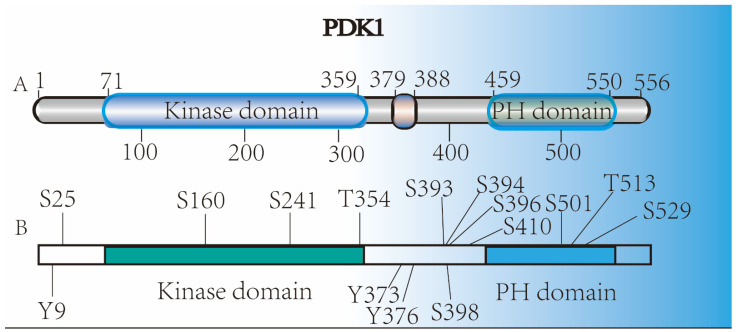
Structure of PDK1 protein: (**A**) the kinase domain (71–359 AAs), the PH domain (459–550 AAs), and the nuclear export sequence (NES) (379–388 AAs) are verified in PDK1 with 556 AAs. (**B**) The phosphorylation sites of PDK1.

As a member of the AGC kinase family, PDK1 was initially discovered in rabbit skeletal muscle in 1997 [7] (shown in Figure 1), and then identified in human 293 cells [8], which were verified to be encoded by human *PDK1* (aliases: *PDPK1*, *PDPK2*, *PDPK2 P*, *PRO0461*; gene ID: 5170; located at 16 p13.3; including 17 exons; approximately 65 kDa; https://www.ncbi.nlm.nih.gov/gene, accessed on 1 September 2021) in 1997 [8]. Being a medium-sized and soluble spherical kinase, PDK1 is a cytosolic protein of 556 amino acids (AAs) composed of two well-documented functional domains: the N-terminal catalytic core domain (the T- or activation loop) and the C-terminal pleckstrin homology (PH) domain (shown in Figure 2). Comparatively, PDK1 does not contain any HM but displays a unique PDK1-interacting fragment (PIF) pocket in the kinase domain, which is separated from the ATP- and substrate-binding sites in the kinase domain. Hence, PDK1 was unambiguously identified not only to be responsible for activating AKT by phosphorylating Thr308 T-loop residue—as well as being the major T-loop kinase for other AGC kinases, such as S6K (S6 kinase beta), RSK (ribosomal S6 kinase), SGK (Serum/glucocorticoid-regulated kinase), and many PKC (Protein kinase C) isoforms—but also to interact with the PIF of protein kinase C-related kinase-2 (PRK2) through the PIF pocket, serving serves as a “docking site” in PDK1 and enhancing the phosphorylation of its substrates [9,10]. Interestingly, a second hydrophobic PIF pocket in the kinase domain of PDK1 was also observed to mediate the interaction and phosphorylation of S6K1 by promoting the binding of S6K1 with PDK1 [11]. On the other hand, as the membrane-targeting site of PDK1, the PH domain was verified to not only specifically interact with the two messengers, PtdIns(3,4,5)P3 (PIP3) and PtdIns(3,4)P2, which are the products of PI3 K, but also directly bind a novel scaffold protein, an adhesion molecule with IgG-like domain 2 (AMIGO2), at residues 465–474 [12,13].

**Figure 3:**
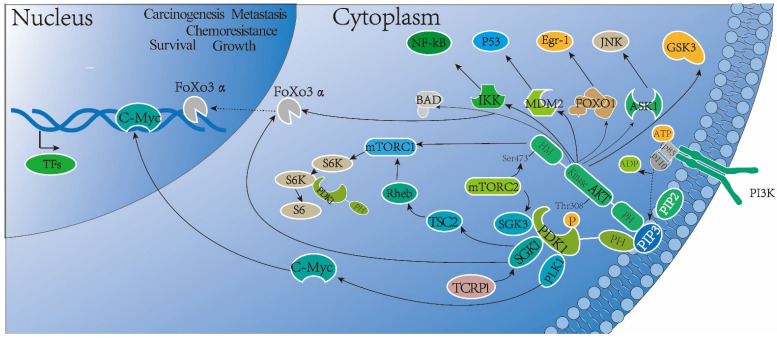
The classical PIP3/AKT-dependent manner of PDK1 activation pathway in BC. Elevated PIP3 induced by growth factors or hormones recruits PDK1 and AKT and simultaneously binds with them through their PH domains, which enhances the interaction between the two allosteric proteins, resulting in a conformational change in AKT and PDK1. This dynamic change makes AKT more prone to phosphorylation at the site Thr308 by PDK1, which is modulated by the rictor–mTOR complex (mTORC2) via phosphorylating AKT on Ser473 of the HM.

Generally, there are two patterns involving PDK1 activation in cellular behaviors [14,15]. Classically, the increased PIP3 stimulated by growth factors or hormones switches on the restrictedly catalytic activity of PDK1 in an inducible manner by the aforementioned PIP3 binding; then, PDK1 is capable of phosphorylating and activating substrates, such as PDK1-mediated AKT activation. Due to the PH domain being possessed by both PDK1 and AKT, simultaneous PDK1 and AKT selective binding with PIP3 has a dual influence on fostering the interaction between the two allosteric proteins and initiates a conformational change in AKT and PDK1, making AKT more prone to phosphorylation on Thr308 of the T-loop by PDK1, which is regulated by the rictor–mTOR complex (mTORC2) via phosphorylating AKT on Ser473 of the HM [16,17,18]. This process is referred to as the canonical PIP3/AKT-dependent manner of PDK1 activation (shown in Figure 3).

**Figure 4:**
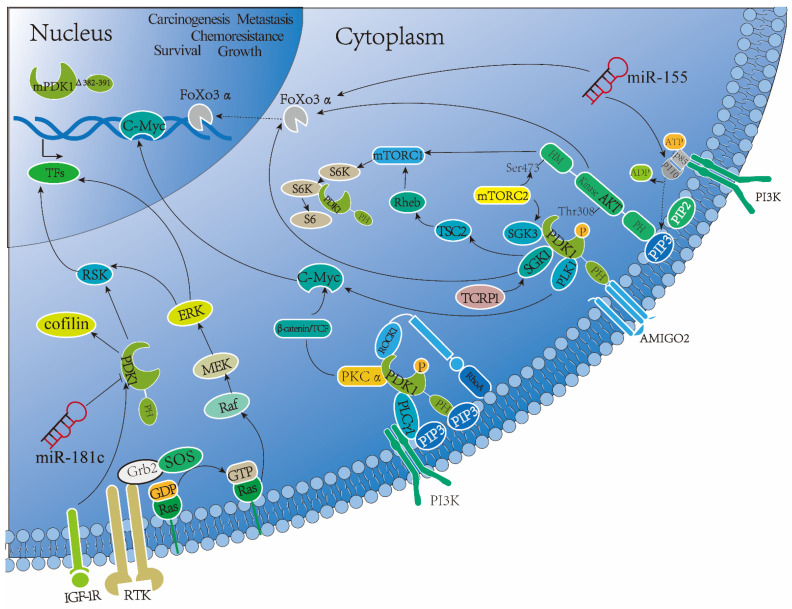
The non-canonical manners of PDK1 activation pathways in BC. Firstly, AMIGO2 interacted with PDK1 by directly binding with PH domain to govern BC cell survival and angiogenesis via activating the AKT pathway. Secondly, PDK1 was proven to be a cytoplasmic–nuclear shuttling protein stimulated by IGF-1 or PTEN loss in the form of mPDK1Δ382–391. Moreover, nuclear PDK1 colocalizing with AKT, which inhibited FOXO3, a transcriptional activity with nuclear localization, was further proved to not only strengthen nuclear pAKT expression and downregulate the transcription of p27 Kip1 in FOXO3α-dependent pattern but also enhance cell growth and proliferation of BC cells. In terms of the latter, activated IGF-1 R/PDK1 signaling was described to reinforce the MCF-7 cells’ growth in nude rats stimulated by light at night, which could be blocked by depleting PDK1. In addition, PDK1 was involved in modulating ROCK1 to impair the cortical actin organization and cell motility of MTLn3 cells, in which PDK1 depletion was responsible for the competitive binding of RhoE to ROCK1. Furthermore, activated PDK1 was described to not only facilitate *KRAS*/*PIK3 CA* comutation-driven carcinogenesis in MCF10 A cells or *MYC*-driven oncogenic transformation of immortalized human mammary epithelial cells via PDK1/PLK1/MYC signaling, but also promote HRG/ERBB2-induced enhanced transformation of BC cells via AKT/TSC2/mTOR. Noteworthily, PDK1 was reported to regulate EGF-induced PLCγ1 Tyr783 phosphorylation of MDA-MB-231 cells, which was referred to as PDK1-dependent PLCγ1 activation, and the interaction PDK1–PLCγ1 played an important role in cancer cell invasion. Interestingly, the PDK1–SGK1 axis was not only shown to overcome AKT inhibition by activating mTORC1 via directly phosphorylating TSC2 in PI3 Kα inhibitor-resistant BC cells, but was also involved in tamoxifen resistance of MCF-7 cells induced by TCRP1. Finally, miR-181 c was observed to negatively regulate targetable *PDK1* in BC cells via suppressing cofilin phosphorylation, resulting in the disruption of the blood–brain barrier; however, another study reported that PDK1 was involved in promoting glucose metabolism via PIK3 R1-PDK1/AKT-FOXO3, a pathway regulated by miR-155.

Alternatively, PDK1 can be activated in various non-canonical manners (shown in Figure 4). In this setting, the aforementioned observations indicate that the PIF pockets of PDK1 are mainly responsible for the phosphorylation of substrates (PRK2 and S6K1) that lack a PH domain [10,11]. Intriguingly, the fact that PDK1 phosphorylated and activated p70 S6 kinase at Thr252 [19], SGK at Ser422 [20], and p90 RSK at Ser227 [21], respectively (which were verified to be inactive by disrupting the PIF pocket of PDK1 in embryonic stem cells [22]) have further illustrated that PDK1 activity could be monitored in such a PIP3-independent manner. Growing evidence indicates PDK1 can also be phosphorylated on tyrosine (Tyr) residues including Tyr-9/373/376 phosphorylation sites, resulting in increased activity. In fact, PDK1 activity was regulated by peroxovanadate through the phosphorylation of all three tyrosines (Tyr-9/373/376) or by reversible tyrosine phosphorylation on Tyr-373/376 rather than Tyr-9 phosphorylation sites, or by Pyk2 in response to angiotensin II, which led to the sequential tyrosine phosphorylation on Tyr-9 and Tyr-373/376 [23,24,25]. In addition, AMIGO2 directly interacts with the PH domain of PDK1 in a non-PIP3-dependent fashion to govern cell survival and angiogenesis via activation of the AKT pathway (shown in Figure 4) [13]. More importantly, PDK1 was observed to induce self-activation through trans-autophosphorylation on Ser241 of the T-loop constitutively [26].

In view of the ubiquitous expression of PDK1 in cells, even in fungi and plants, the fact that PDK1 has unceasingly existed across long-term evolution in a number of living organisms shows PDK1 to be a pivotal player in the progression and function of eukaryotic life [27]. There is increasing evidence emerging in favor of the fact that PDK1 not only exhibits typical translocation from the cytosol to the plasma membrane with activated membrane localization [13,28] but also that it translocates from the cytoplasm to the nucleus stimulated by IGF-1 or PTEN loss, or vice versa, via a series of nucleus-translocation-related functional proteins (termed as nuclear export signal, NES) attributed to a hydrophobic nuclear output sequence of PDK1. Videlicet PDK1 is a cytoplasmic–nuclear shuttling protein, and the fact that PDK-1 nuclear retention is blocked by the PI3 K inhibitors suggests that the cytoplasmic–nuclear shuttling process of PDK-1 may be PI3 K-dependent (shown in Figure 4) [29,30]. Moreover, nuclear colocalization of PDK1 and AKT, which potentially led to the inhibition of FOXO3α transcriptional activity and nuclear localization [30], was further proven to upregulate nuclear pAKT and downregulate p27 ^Kip1^ expression (the gene encoding p27 Kip1 was transcribed in FOXO3α-dependent manner) via antiapoptotic signaling, as well as exhibiting excellent potentiation of cell growth and proliferation [31]. Combined with these results and another discovery that, in human prostate tumors, cytoplasm-localized PDK1 was correlated with early, low-risk tumors, whereas PDK1 nuclear localization was associated with high tumor staging, the presence of solid tumor formation in mice induced by cells with nuclear-localized PDK1 indicated that nuclear translocation of PDK1 mediated oncogenesis and tumor progression [31]. In resting human BC MDA-MB-231 cells, the inactive PDK1 and AKT were mainly distributed in the cytoplasm. Protein translocation of PDK1 from the cytosol region to the plasma membrane was confirmed in MDA-MB-231 cells in the presence of EGF, accompanied by the co-translocation of AKT and PKCζ [32]. Furthermore, the number of BC cells in which EGF-induced AKT translocation occurred was significantly reduced by knockdown of PDK1. Meanwhile, a decreased and delayed PKCζ phosphorylation was detected by knockdown of PDK1 in these EGF-stimulated BC cells compared with control cells [32]. Moreover, knockdown of PDK1 impaired EGF-induced cell adhesion and actin polymerization in BC cells, as well as tumorigenesis and lung metastasis of BC cells in severe combined immunodeficient (SCID) mice. Therefore, it is speculated that PDK1 might modulate the migration and experimental metastasis of human BC cells [32]. Additionally, recent data showed that continued AKT-targeted therapies in luminal BC cells elicited dephosphorylation and nuclear translocation of FOXO3a, and impaired the link between FOXO3a and SirT6, ultimately leading to acetylated FOXO3a, which recognized the BD2 domain of BRD4, recruited the BRD4/RNAPII complex to the CDK6 gene promoter, and induced its transcription. Chemical interference with either BRD4/FOXO3a or CDK6 markedly re-sensitized luminal BC cells to AKT inhibitors in vitro and in vivo (shown in Figure 4) [33]. Unfortunately, it is currently unclear whether or how nuclear-localized PDK1 may affect the developmental or treatment events of BC cells by regulating AKT and FOXO3a. As such, more comprehensive research may be conducive to exploring the potential roles of nuclear-localized PDK1 in BC cells via the PI3 K/AKT pathway.

## 3. The Roles of PDK1 in Carcinogenesis, Cancer Growth, and Survival of BC Cells

It is well established that the disruption of the normal autoinhibitory constraints on kinase activity involved in monitoring growth and survival could cause the malignant transformation of cells [34]. As a moderator of various signaling pathways closely related to growth factor receptor activation, the oncogenic role of PDK1 was evaluated in COMMA-1 D cells (mouse mammary epithelial cells). A high degree of transformation was displayed in PDK1-expressing cells accompanied by activated AKT1 and over-expressed PKCα. Anchorage-independent growth was not observed in AKT1-overexpressing cells, whereas PKCα overexpression yielded marked transformation, albeit to a lesser degree compared with PDK1. Coexpression of AKT1 and PKCα created a more than additive effect on the transformation activity of cells. The formation of poorly differentiated BCs was convincingly observed in syngeneic mice tumor grafts with either PDK1- or PKCα-expressing cells, but not AKT1-expressing cells. Elevated expression of PDK1 was proved in a majority of human BC cell lines [35]. These results were suggestive of the PDK1-mediated transformation of mammary epithelial cells via PKCα but not AKT1 downstream. An oncogenic pathway downstream of PDK1 and PKCα that was linked to overexpression of c-Myc and cyclin D1 through activation of the β-catenin and PKC promoter, as well as suppressing caveolin-1 in mammary epithelial cells, was further confirmed in the follow-up research [36]. In fact, although the data that PDK1 expression was moderate to strong in BC specimens (213/241, 88%) coincided with the findings in BC cells [35,37], PDK1 overexpression was not correlated with the mutation status of *PIK3 CA* in BC specimens, suggesting that PDK1 could be independently activated in BC and not just as a part of the PIK3 CA signaling pathway [37]. In agreement with these findings, Maurer et al. showed overexpression of total PDK1 protein (50/69, 72%) and mRNA, as well as *PDK1* gene copy number increase (27/129, 21%) in human BC samples [38]. Of note, a *PDK1* copy number increase was associated with *PIK3 CA* mutation, as well as patient survival [38]. More importantly, to determine the biological effects of PDK1 overexpression on ERBB2-induced transformation in vitro and tumor growth in vivo, a set of four human mammary epithelial cell lines MCF10 A were generated by transfection of retroviral vectors expressing PDK1 (+PDK1), expressing ERBB2 (+NeuT), expressing both (+PDK1 +NeuT), or control vectors. The result was that morphological changes including distorted multiacinar structures and cell foci coupled by interconnecting branching tracts, were shown in +PDK1 +NeuT MCF10 A cells, which shortly led to large muscle-invasive tumors in all SCID mice, indicating that PDK1 overexpression impelled ERBB2-induced transformation and tumor growth [38]. Similarly, the activated PDK1 displayed an especially important role either in carcinogenesis of MCF10 A cells induced by the concurrent mutations in both *KRAS* and *PIK3 CA* [39], or for the process of HRG/ERBB2-induced and enhanced transformation of BC cells (SKBR3, MCF7, and ZR-75–1 cells) via AKT/TSC2/mTOR [40]. Noteworthily, PDK1 was observed to promote MYC-driven oncogenic transformation of not only HEK cells but also immortalized human mammary epithelial cells (HMECs) and prostate epithelial cells via PDK1/PLK1/MYC signaling [41]. Thus, the evidence supporting the conclusion that hyperactive PDK1 potentiates mammary carcinogenesis by mediating the transformation of mammary epithelial cells is sufficient, underlying these diverse views.

As the backbone of cancer cells’ biological events, cancer growth and survival have been extensively confirmed to be related to dysfunctional PDK1 signaling in various cancers, such as renal cell carcinoma (RCC) cells [42], hepatocellular carcinoma (HCC) cells [43], human glioblastoma cells [44,45], melanoma, and colon cancer cells [46]. The fact that PDK1 silencing reduced proliferation in MCF7 and T47 D cells harboring *PIK3 CA* mutation [38], and that both genetic and chemical PDK1 inhibition sufficiently hindered a set of BC cell lines forming soft agar colonies [47], implied the impact of PDK1 in anchorage-independent cell growth. Furthermore, PDK1 was proven to be required for anchorage-independent growth and xenograft formation of BC cells (MDA-MB-231 and T47 D cells) harboring either *PI3 KCA* or *KRAS* mutations attributed to the facts that PDK1 knockdown led to increased anoikis, reduced soft agar growth, and indubitable apoptosis inside tumors, which could be rescued by wild-type PDK1 instead of inactivated mutant PDK1; however, this could not be reverted by constitutively activated AKT in PDK1-silencing cells. Moreover, neither genetic nor pharmacological inhibition of AKT could suppress the effects of PDK1 overexpression [48]. In addition, enhanced PDK1 expression was observed to be correlated with excessive metabolic activation in a spontaneous PyMT-induced BC mice model, and either pharmacological or genetic inhibition of PDK1 could overwhelm BC cell growth in vitro [49]. Intriguingly, a study reported that light at night activated IGF-1 R/PDK1 signaling and accelerated tumor growth of MCF-7 cells in nude rats, which could be inhibited by PDK1 knockdown [50].

In essence, these discoveries suggest that PDK1 contributes to the cancer growth and survival of BC cells, even without PI3 K oncogenic mutations, in either an AKT-dependent or AKT-independent fashion, which identifies PDK1 as a potential therapeutic target for BC. However, the above evidence seems to be less convincing than the evidence coming from the following investigations regarding the role of PDK1 in BC metastasis to support PDK1 as a potential targeted therapy in BC owing to the evidence that relatively delayed BC lesions are ultimately generated in a handful of PDK1-deficient tumorigenic cells [49], as well as the evidence that PDK1 harnesses migratory and oncogenic transforming behaviors rather than growing and proliferative phenotypes in PTEN-loss lymphocytes [51]. Further study is warranted to confirm the limited information on this topic in BC cells.

## 4. The Mechanisms of PDK1 in Metastatic Spreading of BC Cells

Metastasis, the leading cause of BC-related deaths worldwide, is the multistage process of BC cells propagating from a primary site to progressively colonize distant organs through blood vessels/lymphatic vessels. It consists of four steps: detachment, migration, invasion, and adhesion [52,53,54]. Evidently, this complex succession of a spectrum of cell-biological events, termed the “invasion–metastasis cascade”, is closely related to tumor microenvironment (TME) regulation, which comprises extracellular matrix structure, growth factors, chemokines, matrix metalloproteinases, immune cells, etc. [52]. Therefore, we reviewed recent insights into the molecular understanding of the mechanisms of PDK1 in BC metastasis and inferred that dysregulated PDK1 signaling promoted BC metastasis.

Firstly, elevated phosphorylation and activation of PDK-1 pSer241 were observed not only in BC cell lines (non-invasive MCF-7 and invasive MDA-MBB-468 cells) but also in invasive BCs (72/89, 80.9%), particularly in high-grade metastatic BCs (18/21, 86%) [55]. Consistent findings were obtained in human invasive BC tissue microarray analysis, as a moderate to strong expression of PDK1 pSer241 was observed in 90% of all tumor specimens, in which 42% of evaluable specimens displayed strong expression (74/177 cores) [56]. Moreover, enhanced invasion on Matrigel in PDK1-expressing Comma-1 D mouse mammary epithelial cells (Comma/PDK1 cells, which produced invasive adenocarcinomas in mammary fat pad isografts) was confirmed and accompanied by increased MMP-2 activity resulting from stabilization against proteasomal degradation, as well as accompanied by elevated levels of MT1-MMP; this, in turn, generated active MMP-2 and modulated the ECM proteins decorin and collagen. As such, PDK1 serves as an important effector of cell growth and invasion in the transformed cells [56]. The findings that, as described in detail above, PDK1 overexpression potentiated ERBB2-induced transformation and migration of human MCF10 A cells [38], as well as that PDK1 regulated EGF-induced chemotaxis and directional migration and invasion of MCF10 A cells by binding and activating MRCKα in an AKT-independent manner [57], provide further insight into the influence of PDK1 on metastasis of the transformed cells. As discussed earlier, PDK1 depletion in BC cells inhibited metastasis by reducing EGF-mediated chemotaxis and adhesion of tumor cells and aggregation of actin via the dampening of phosphorylation and translocation of AKT and PKCζ in a kinase-dependent manner, as well as suppressed tumorigenesis and lung colonization in SCID mice [32]. Additional evidence that PDK1 regulated the invadopodia formation of BC cells (MDA-MB-231, BT-549, and Hs578 T cells) via p110α/PDK1/AKT signaling supports the notion that PDK1 is implicated in the metastasis of BC cells in a kinase-dependent manner [58,59]. Interestingly, the depletion of PDK1 disrupted the cortical actin organization and cell motility of MTLn3 cells (highly metastatic mouse BC cells) in vitro and in vivo by regulating ROCK1 in a novel AKT-independent mechanism, in which ROCK1 was associated with RhoE in the absence of PDK1 [60]. In fact, PDK1 regulated the EGF-induced PLCγ1 activation of MDA-MB-231 cells in a different AKT-independent manner, and the interaction of PDK1–PLCγ1 was proved to be important for cancer cell invasion (shown in Figure 4) [61]. PDK1/PLCγ1 interaction related to migration and invasion of BC cells was further established to be blocked by a small molecule inhibitor, 2-O-Bn-InsP5, in the following study [62]. The GENEOM clinical trial (ClinicalTrials.gov Identifier: NCT00959556) involved collecting blood samples from 342 BC patients and investigating 88 SNPs of PI3 K/AKT/mTOR signaling genes (including: 17 SNPs of *AKT1*, 4 of *AKT2*, 2 of *FGFR1*, 7 of *mTOR*, 4 of *PDK1*, 11 of *PI3 KR1*, 20 of *PI3 KCA*, 17 of *PTEN*, and 6 of *RPS6KB1*). This study exhibited not only the association between *PI3 KR1*-rs706716 and central nervous system (CNS) metastasis by univariate analysis but also the associations between *AKT1*-rs3803304, *AKT2*-rs3730050, *PDK1*-rs11686903, and *PI3 KR1*-rs706716 and CNS metastasis by multivariate analysis [63].

Apart from the effect of PDK1 deletion on T cell lineage development [64,65], PDK1 was further proved to overwhelm metastasis of BC by regulating the immune cells of TME in a PyMT-induced BC mice model by mediating macrophage polarization [49,66]. Myeloid-specific inactivation of PDK1 reprogrammed the metabolism of tumor-infiltrating macrophages and stimulated M1 macrophage polarization by inhibiting the mTOR pathway, while also retarding tumor growth and suppressing lung metastasis of BC model [66]. 

It is generally accepted that the overwhelming majority of cancers demonstrated organ-specificity of metastasis, a phenomenon known as “organotropism” [67]. To improve the prognosis of metastatic BC, a deeper understanding of the mechanisms by which BC metastasizes, particularly those latent organotropisms toward the brain, bone, lungs, and liver, is imperative [68]. Luminal BCs, in particular, tend to metastasize to bone, while BLBCs display organotropic metastasis to the lung. Great amounts of data have revealed that exosomal microRNAs mediate BC metastasis to preferential premetastatic niches: brain, lungs, and bone. For instance, miR-181 c, miR-19a, miR-503, and miR-105 are involved in the brain metastasis of BC [69,70,71,72,73,74,75]; miR23 b, miR940, miR20 a-5p, and miR127 are associated with bone metastasis [76,77]; while miR105, miR202, miR-200 a-c, and miR210 mediate BC metastasis to the lung [76]. Regarding the fact that PDK1 facilitated tumor cell migration and infiltration into the lungs by regulating epithelial–mesenchymal transition (EMT) [49], as well as the fact that miR-155 harnessed the tumor growth of BC cells in vivo via PIK3 R1-PDK1/AKT-FOXO3a pathway [78], a study revealed that miR-181c boosted brain metastasis of BC cells via directly downregulating *PDK1* to destroy the blood–brain barrier (shown in Figure 4) [69], which compelled us to hypothesize that PDK1 might take part in the organotropic metastasis of BC. We believe this finding could develop a novel perspective for molecular mechanism research regarding PDK1 in BC metastasis.

In summary, PDK1 was identified as a potential therapeutic target for the role of PDK1 in BC metastasis.

## 5. The Influence of PDK1 on Drug Resistance in BC Cells

Owing to its essential role in metastatic potential, transformation induction, and cell fate determination, it is not surprising that aberrant PDK1 plays a part in drug resistance of BC. Numerous findings have documented that hyperactive PDK1 signaling reinforces BC chemoresistance by various means. Given the fact that there were considerable diversities in the expression and activation-specific phosphorylation levels of PDK1 and AKT1 among the BC cell lines, and the fact that the phosphorylation of PDK1 or AKT1 was related to the sensitivity of BC cells to chemotherapy drugs, cells highly expressing Ser241-phosphorylated PDK1 (MDA453 and SKBR3 cells) were shown to be more resistant to gemcitabine than cells exhibiting high levels of Ser473-phosphorylated AKT1 (MDA468 and MDA231 cells). Furthermore, PDK1 blockade re-sensitized MCF7 cells to gemcitabine more efficiently than AKT1 ablation. Thus, much attention has focused on PDK1, which might be a superior substitute for AKT1 as a target against resistance of BC cells to gemcitabine [79]. Analogously, PDK1 was affirmed to be a key modifier of acquired resistance to CDK4/6 inhibitors in ER-positive BC cells via the PI3 K/PDK1 pathway in both AKT-dependent and AKT-independent manners, and the combination of CDK4/6 inhibitor ribociclib or palbociclib and PDK1 inhibitor GSK2334470 synergistically suppressed proliferation and increased apoptosis in several ER+ BC cell lines in vitro and in vivo [80]. Additionally, the PI3 K–PDK1–AKT^T308^ signaling axis was reported to sustain the survival of lapatinib-resistant BC cells (rBT474) [81].

Since SGK1 was verified to have a role in BC cells’ resistance to AKT inhibitors under PDK1 regulation [82], the PDK1–SGK1 axis was further revealed to prevail against AKT inhibition by activating mTORC1 via directly phosphorylating TSC2 in BC cells resistant to PI3 Kα inhibitors, while PDK1 deletion re-sensitized BC cells to PI3 Kα inhibitors [83]. Indeed, aside from the fact that the PDK1 signaling pathway was confirmed as a strong determinant of sensitivity to tamoxifen [84], TCRP1 was observed to induce tamoxifen resistance by modulating the PDK1/SGK1 signaling pathway in MCF-7 cells (shown in Figure 4) [85]. We thereby inferred that the PDK1–SGK1 axis displayed an especially important role for an acquired resistance mechanism in BC cells, which makes it a promising candidate in targeted therapeutics of BC.

As noted, PDK1 not only induced oncogenic transformation but also evoked a CSC-like phenotype via PDK1–PLK1–MYC signaling in MDA-MB-231 cells, which led to resistance to mTOR-targeted therapy [41]. Interestingly, the multicenter research mapped the phospho-catalytic profile of kinases of the drug-resistant tumors by screening 228 peptides and revealed that activated PDK1 mediated the intrinsic resistance to BRAF^V600 E^-targeted therapy in colorectal cancer (CRC). Although the study employed BRAF^V600 E^-driven tumors as a test scenario, we hope this new approach could identify the druggable vulnerabilities in BC [86].

Collectively, as emerging data have started to uncover its underestimated impacts on drug resistance, there are increasing expectations that encouraging results of preclinical or clinical studies on targeted PDK1 could be achieved in chemoresistant BC cells, or even better.

## 6. Targeted Therapies of PDK1 Signaling in BC

Over the past three decades, numerous studies on the role of PDK1 in cancers have yielded important results that have paved the way for the development of anti-PDK1 monoclonal antibodies and specific or nonspecific PDK1 inhibitors. Nevertheless, so far, no selective PDK1 inhibitor has been available for BC therapy. We list the preclinical or clinical trials of PDK1-targeted therapy in BCs according to their groups in Table 1 [41,47,48,58,61,62,80,83,87,88,89,90,91,92,93,94,95,96,97] and extract the important findings (as of September 2021, http://clinicaltrials.gov, accessed on 1 September 2021).

UCN-01, the first identified Chk1 inhibitor, was recognized as a nonselective inhibitor of PDK1, which was proved to have a potential hydrogen-bonding residue at the site coupled to Thr222 T-loop of PDK1 [98,99,100]. In 1993, UCN-01 was initially observed to inhibit the growth of five BC cell lines (MCF-7, MDA-MB453, SK-BR-3, H85787, and MDA-MB468) and induce cell cycle arrest in [89], and then the maximum tolerated dose (MTD) of UCN-01 was estimated in patients with refractory BCs from 1995 to 2002 (ClinicalTrials.gov Identifier: NCT00001444). Furthermore, the MTD and side effects (SEs) of UCN-01 and irinotecan hydrochloride in patients with metastatic, unresectable, or resistant BCs were evaluated, starting in 2001 (ClinicalTrials.gov Identifier: NCT00031681). Interestingly, UCN-01 was affirmed to potentiate camptothecin (CPT)-induced cytotoxicity in two human BC cell lines with a mutated *p53* gene via modulating CPT-activated S and G2 checkpoints, which indicated the potential efficacy of combined therapy with UCN-01 and topoisomerase I inhibitors in BCs with an aberrant *p53* gene [90]. Additionally, based on the beneficial combination therapy of UCN-01 and irinotecan in p53-loss triple negative breast cancer (TNBC) in vivo with xenograft mice models [91], Ma et al. further executed a phase II clinical trial of combination therapy of UCN-01 and irinotecan in 25 patients with metastatic TNBC and found that effective UCN-01 could enhance irinotecan-induced apoptosis in *TP53*-mutant BCs (69% of tumors were basal-like BCs, BLBCs); they also illustrated the molecular heterogeneity of TNBC and correlated the relevance of *TP53*-mutant TNBC to a pretty poor survival. These results suggested a need to classify the subjects in clinical trials [92]. Surprisingly, a recent study disclosed that pre-treatment with UCN-01 in preclinical models (non-tumor-bearing and MDA-MB-468 tumor-bearing mice) prior to 5-FU potentiated therapeutic efficacy with remarkable shrinkage of tumors and increased survival [93]. In short, these findings produced a cornerstone for future clinical trials of UCN-01 in TNBC.

In a patent published in 2010, GSK2334470 was originally described as a highly specific inhibitor of PDK1 [101], which was then characterized as more specific than UCN-01 or BX-795 in 2011 [102]. GSK2334470 not only suppressed PDK1 from activating full-length AKT1 stimulated by PIP3-containing lipid vesicles or a mutant AKT1 in the absence of PH domain but also blocked the phosphorylation of PDKtide activated by PDK1 [102]. As mentioned above, PDK1 regulated EGF-induced PLCγ1 activation of MDA-MB-231 cells, and therapy with GSK2334470 completely eliminated the increased EGF-induced intracellular calcium and accumulated inositol phosphates, as well as inhibiting PLCγ1 Tyr783 phosphorylation and invasive phenotype in MDA-MB-231 cells [61]. Moreover, the evidence that PDK1 deletion with GSK2334470 re-sensitized BC cells to PI3 Kα inhibitor BYL719 [83] and CDK4/6 inhibitors, respectively [80], was encouraging enough to merit further clinical trials of GSK2334470 in patients with metastatic or acquired resistant BCs.

Notwithstanding that a new antibody targeting extracellular PDK1 was patented in 2016 [89,103], treatment with anti-PDK1 monoclonal antibodies in BC cells or patients has not been reported until now. However, the combination therapy trials of PDK1-targeted therapy and other agents have provided some benefit. With emerging creations and synthesis of PDK1 inhibitors [89], more alternatives of PDK1-targeted therapy are becoming available for exploration in BC.

## 7. Discussion

Evidently, as a key protein kinase at the crossroad of AGC signaling pathways, PDK1 is inevitably a promising target with multiple roles in BC, particularly for those tumors with aggressiveness, metastasization, and chemoresistance. Despite numerous attempts having been made in BC therapy with candidate PDK1 tool compounds, definitive evidence about whether specific inhibitors of PDK1 as monotherapy could achieve favorable results is still lacking; this obliges us to take actions to determine the vulnerabilities of the therapeutic potential in specific PDK1 inhibitors. Fortunately, besides the promotion of PDK1 inhibitor invention and development, several studies also established the therapeutic potential of PDK1 inhibitors in combination with other agents in BCs. However, the clinical benefit of PDK1 inhibitors in BCs is not as impressive as that of the other PI3 K/AKT signaling inhibitors at present. It is imperative to develop more innovative options for a targeted PDK1 treatment strategy in BCs. As noted above, the involvement of PDK1 in T cell lineage development [64,65] makes it possible to combine PDK1-targeted therapy with immunotherapy in BCs, such as immune checkpoint inhibitors (ICIs) and anti-PD1/PDL1 therapy. The clinical feasibility, safety, and potential effectiveness of combining PDK1 inhibitors in anti-PD1/PDL1-based drug therapy in BCs deserve further verification, which might lead to breakthroughs in BC treatment. Accordingly, the view that drug combination therapies of PDK1 inhibitors give rise to some clinical efficacy in BCs will likely be accepted gradually in the coming years.

MicroRNAs (miRNAs) are a class of small, conservative, and non-coding RNAs that regulate more than 30% of coding genes by binding to the 3′ end of a target RNA [104]. In recent years, plentiful miRNAs have been described as being involved in the malignant phenotype of BC cells, such as carcinogenesis, proliferation, invasion, EMT, stemness, therapy resistance, as well as aforementioned metastasis [69,78,105,106,107,108,109,110,111]. However, microRNA-based regulation of PDK1 is rare in BC. Only miR-181c was shown to negatively regulate targetable *PDK1* in brain metastasis of BC cells [69]. Kim et al. described how miR-155 directly regulated *PIK3 R1* and *FOXO3α*, not *PDK1*, to promote glucose metabolism via the PIK3 R1-PDK1/AKT-FOXO3α pathway [78]. More studies focusing on microRNA-based regulation of PDK1 are promising and have the potential to improve the therapeutic approaches whilst addressing the root causes of BC metastasis.

## 8. Conclusions

Overall, PDK1, which was previously underestimated, has now been uncovered to possess an attractive influence on the biological behavior of BC cells, particularly in carcinogenesis, proliferation and survival, metastasis, and drug resistance. Targeted PDK1 therapy has achieved some benefit in BCs, and we mapped the latest progression of PDK1 in BC to the clinic and wish to bring about advantageous changes in treatment for patients.

## Figures and Tables

**Table 1:** Preclinical and clinical trials of PDK1-targeted therapies in breast cancers.

Class	Type	Inhibitor	Characteristic	Inhibition Mode	Identifier and Phase of Clinical Trials	Patent Date /Publication Date /Clinical Trials Start Date	Patent Application Number/ References
Heterocyclic compounds	Selective inhibitor	Compound 7	Compound 7 impaired anchorage-independent growth, invasion, and cancer cell migration of BC cell lines.	T-loop phosphorylation		25 February 2011	[47]
		GSK2334470	Therapy with GSK2334470 completely eliminated the increased EGF-induced intracellular calcium and accumulated inositol phosphates, as well as inhibited PLCγ1 Tyr783 phosphorylation and invasive phenotype in MDA-MB-231 cells.	T-loop phosphorylation		1 July 2012	[61]
			PDK1 deletion with GSK2334470 re-sensitized BC cells to PI3 Kα inhibitor BYL719.			8 August 2016	[83]
			Inhibition of PDK1 with GSK2334470 resensitized ribociclib-resistant cells to CDK4/6 inhibitors and the combination of CDK4/6 inhibitor ribociclib or palbociclib and GSK2334470 synergistically suppressed proliferation and increased apoptosis in several ER+ BC cell lines in vitro and in vivo.			1 May 2017	[80]
		PHT-427	Combination studies showed that PHT-427 has greater than additive antitumor activity with paclitaxel in MCF-7 BC xenografts.	PH domain		1 March 2010	[87]
		2-*O*-Bn-InsP5	2-*O*-Bn-InsP5 interacted specifically with the PH domain of PDK1 and impaired formation of a PDK1/PLCγ1 complex, ultimately blocking BC cell invasion.	PH domain		20 May 2016	[62]
		Compound I-IX	The compounds with 443 molecules were classified in 9 substructures, which has indicated applications in BC.	PIF pocket		20 September 2018	AU2018222943 [88,89]
	Nonselective inhibitor	UCN-01	UCN-01 was first observed to inhibit the growth of five breast carcinoma cell lines and induce cell cycle arrest.	T-loop phosphorylation		1 May 1993	[90]
			To estimate the MTD of UCN-01 in patients with refractory BCs.		NCT00001444I	1 August 1995	-
			Determine the MTD and side effects of UCN-01 and irinotecan hydrochloride in patients with metastatic, unresectable, or resistant BCs.		NCT00031681I	1 December 2001	-
			The study investigated the ability of UCN-01 to potentiate CPT-induced cytotoxicity in two human BC cell lines with mutated p53 gene via modulating CPT-activated S and G2 checkpoints and found the potential efficacy of combined therapy with UCN-01 and topoisomerase I inhibitors in BCs with an aberrant *p53* gene.			1 February 2000	[91]
			The study reported that combination therapy with UCN-01 and irinotecan induced checkpoint bypass and apoptosis, as well as inhibited tumor growth and prolonged survival in p53-deficient TNBC tumor models.			2 April 2012	[92]
			The study showed that effective UCN-01 could enhance irinotecan-induced apoptosis in *TP53* mutant BCs and illustrated the molecular heterogeneity of TNBC and the relevance of *TP53* mutant TNBC leading to a pretty poor survival, suggesting a need to classify the subjects in clinical trials.		II	1 January 2013	[93]
			Pre-treatment with UCN-01 in preclinical models (non-tumor-bearing and MDA-MB-468 tumor-bearing mice) prior to 5-FU potentiated therapeutic efficacy with remarkably shrinking tumor and increasing survival.			1 March 2020	[94]
		OSU-03012	OSU-03012 potentiated trastuzumab’s antiproliferative effect in HER2-positive cells, especially in SKBR3/IGF-IR cells, via downregulating PDK-1/AKT signaling.	COX-2 activity		1 November 2006	[95]
			OSU-03012 blocked invadopodia formation of MDA-MB-231 cells.			27 June 2011	[58]
			OSU-03012 blocked anchorage-independent growth of MDA-MB-231 cells and promoted anoikis.			1 August 2012	[48]
		BX-795	BX-795 inhibited soft agar growth of BC cells very effectively.	T-loop phosphorylation		1 August 2012	[48]
			BX-795 treatment resulted in similar MYC depletion in all these cells but preferentially reduced the cell viability of MYC-dependent breast cancer cell lines (MDA-MB-231, Hs578 T, and SUM159 PT) as compared with the MYC-independent breast cancer cell lines (T47 D and BT474). Moreover, BX795 treatment resulted in marked inhibition of tumorsphere formation in MDA-MB-231 cells.			1 October 2013	[41]
		BX-912	BX-912 treatment resulted in similar MYC depletion in all these cells but preferentially reduced the cell viability of MYC-dependent breast cancer cell lines (MDA-MB-231, Hs578 T, and SUM159 PT) as compared with the MYC-independent breast cancer cell lines (T47 D and BT474). Moreover, BX912 treatment resulted in marked inhibition of tumorsphere formation in MDA-MB-231 cells.	T-loop phosphorylation		1 October 2013	[41]
Pyridonyl derivatives	Nonselective inhibitor	Pyridons	Pyridons was found to be effective against MDA-MB-468 cells, in which the PI3 K/AKT/PDK1 signaling pathway is up-regulated.			1 November 2011	US0269958, [96]
		Pyridons	Pyridons were tested for cell growth/death in several human BC cell lines (BT474, HCC1954, T-47 D). There were no reports on in vivo data or selectivity, despite their potent in vitro activity.			1 May 2010	WO019637, [97]

BC, breast cancer; CPT, camptothecin; MTD, maximum tolerated dose; TNBC, triple negative breast cancer. Clinical Trials Identifiers NCT00001444 available at https://clinicaltrials.gov/ct2/show/NCT00001444 (accessed on 1 September 2021) and NCT00031681 available at https://clinicaltrials.gov/ct2/show/NCT00031681 (accessed on 1 September 2021).

## Data Availability

The data presented in this study are available within the article.

## Abbreviations

AAs: amino acids; AMIGO2, adhesion molecule with IgG-like domain 2; BC, breast cancer; BLBC, basal-like breast cancer; CNS, central nervous system; CPT, camptothecin; DCP, des-gamma-carboxyl prothrombin; EMT, epithelial–mesenchymal transition; ER, estrogen receptor; HCC, hepatocellular carcinoma; HER2, human epidermal receptor 2; HM, hydrophobic motif; HMEC, human mammary epithelial cells; IBC, invasive breast carcinoma; IDC, invasive ductal carcinoma; ICIs, immune checkpoint inhibitors; MTD, maximum tolerated dose; mTORC2, rictor–mTOR complex; NOS, not otherwise specified; NES, nuclear export signal; NST, no special type; PDK1, 3-phosphoinositide-dependent protein kinase 1; PH, pleckstrin homology; PI3 K, phosphatidylinositol 3-kinase; PIF, PDK1-interacting fragment; PIP3, PtdIns(3,4,5)P3; PKCα, protein kinase C α; PR, progesterone receptor; PRK2, protein kinase C-related kinase-2; RCC, renal cell carcinoma; SCID, severe combined immunodeficient; SE, side effect; TME, tumor microenvironment; TM/Z, turn motif or zipper site; TNBC, triple negative breast cancer; Tyr, tyrosine; WHO, World Health Organization.
